# A wearable IMU-based framework for daily physical activity recognition and energy expenditure level classification in university students

**DOI:** 10.3389/fpubh.2026.1829967

**Published:** 2026-05-28

**Authors:** Xiaoming Zhou, Shiping Wang, Lei He, Li Ma

**Affiliations:** 1 School of Journalism and Communication, Beijing Sport University, Beijing, China; 2 Department of Physical Education, Woosuk University, Wanju-gun, Jeollabuk-do, Republic of Korea; 3 School of Physical Education, Chizhou University, Chizhou, China

**Keywords:** energy expenditure, human activity recognition, inertial measurement unit, machine learning, physical activity, university students, wearable sensors

## Abstract

**Introduction:**

Insufficient physical activity and prolonged sedentary behavior remain common among university students, yet conventional self-reports suffer from recall bias. The key idea of this research is to establish a unified, single-sensor framework that bridges engineering-based activity recognition with public health-oriented energy assessment by mapping raw inertial signals directly to metabolic intensity levels.

**Methods:**

This study developed and evaluated a waist-worn IMU-based framework for daily physical activity recognition and energy expenditure level classification. A purpose-built dataset was collected from 96 healthy university students (mean age 20.4 ± 1.7 years), yielding 106,376 valid signal windows across six representative activities: sitting, standing, level walking, stair ascent, stair descent, and jogging. Triaxial acceleration and angular velocity signals were processed through filtering, normalization, and sliding-window segmentation.

**Results:**

Under leave-one-subject-out (LOSO) validation, the best-performing CNN-LSTM model achieved an accuracy of 93.8% (macro-F1: 0.932) for activity recognition and 89.6% (macro-F1: 0.883) for three-level energy expenditure classification.

**Discussion:**

The primary contribution lies in validating single-sensor feasibility within a specific campus movement ecology, demonstrating that the system can simultaneously deliver behavioral and metabolic insights without *post hoc* label mapping. This study fills a critical knowledge gap by quantifying the accuracy trade-offs of a joint pipeline under rigorous subject-independent conditions, providing a feasible basis for large-scale campus health surveillance.

## 1 Introduction

Regular physical activity is closely associated with physical and mental health, whereas insufficient activity and prolonged sedentary behavior are linked to unfavorable cardiometabolic, psychological, and functional outcomes (1). Current public health recommendations emphasize the dual importance of accumulating adequate moderate-to-vigorous physical activity and reducing sedentary time in daily life (1). University students represent a population for whom these recommendations are especially relevant. During higher education, daily movement behavior is often shaped by prolonged classroom attendance, extensive screen-based learning, irregular schedules, commuting demands, and unstable exercise routines. As a result, insufficient physical activity and high sedentary exposure remain widespread in university settings (2, 3). Objective measurement studies have found that university students accumulate approximately 8.4 h of sedentary time per day on average, with only around half meeting established moderate-to-vigorous physical activity guidelines (2, 4, 5). These behavioral patterns are associated with elevated risks of cardiometabolic disease and diminished psychological well-being, highlighting the public health relevance of monitoring student activity with sufficient precision (1, 2).

A longstanding challenge in this field concerns measurement. Questionnaire-based instruments and retrospective activity logs remain common because they are inexpensive and easy to administer at scale. However, self-report measures are susceptible to recall bias, social desirability bias, and poor temporal resolution, and they are often inadequate for identifying short-duration, low-intensity, or context-dependent daily behaviors (6, 7). These limitations are particularly problematic in student populations, whose movement patterns are typically fragmented across studying, classroom sitting, campus walking, stair use, and intermittent exercise. When the research objective extends beyond broad activity volume to the recognition of specific behaviors and their intensity, more objective approaches are needed.

Wearable sensing technologies provide one such approach. Accelerometers and inertial sensors have been widely adopted in physical activity research because they support continuous and relatively unobtrusive monitoring in naturalistic settings (8–10). Among these devices, wearable IMUs are particularly useful because they combine accelerometers and gyroscopes, thereby capturing both translational and rotational characteristics of movement. This makes IMUs suitable not only for estimating activity quantity but also for identifying distinct activity classes and movement patterns (9–14). In recent years, IMU-based monitoring has been applied in gait analysis, exercise evaluation, rehabilitation, sports biomechanics, and free-living daily activity assessment (12–14).

Human activity recognition (HAR) based on wearable sensors has developed considerably over the past decade. Earlier studies mainly relied on carefully engineered time-domain and frequency-domain features extracted from inertial signals and classified with models such as support vector machines, decision trees, and random forests (9, 15–17). Later work showed that even a single body-worn sensor can provide strong recognition performance under appropriate placement and preprocessing conditions (16, 17). The effect of sensor placement on recognition accuracy has received renewed attention in recent work: studies examining IMU positions from wrist to ankle have demonstrated that placement choice systematically affects discriminative performance across activity classes, with trunk-level sensors showing particular stability for locomotion tasks (18). A comprehensive review by Zhang et al. confirmed that deep learning methods have progressively improved HAR performance by learning representations directly from raw sensor streams, with hybrid architectures combining convolutional neural networks (CNNs) and long short-term memory (LSTM) networks showing particular effectiveness on benchmark datasets (19). The CNN component extracts local temporal patterns, while the LSTM component captures sequential dependencies, making such architectures well suited to periodic inertial data (11, 19, 20). Among deep learning architectures, Inception-ResNet variants have demonstrated competitive performance for HAR by enabling multi-scale feature extraction within a single network, with the iSPLInception architecture reported to achieve strong accuracy on benchmark datasets while maintaining computational tractability (21). More recent work has further demonstrated that augmenting CNN-LSTM models with self-attention mechanisms can improve subject-independent generalization, with Khatun et al. reporting accuracies exceeding 93% on the UCI-HAR dataset under cross-subject evaluation (22).

Parallel to advances in activity recognition, interest has grown in inferring energy expenditure from inertial signals alone. Lopes et al. showed that a deep learning model trained on IMU data can estimate steady-state energy expenditure during walking and running without requiring additional physiological instrumentation (23). Subsequent work extended this approach to varied walking speeds and incline conditions, reporting consistent estimation performance with IMU-only input (24). These developments suggest that the combination of activity recognition and MET-informed intensity classification within a single sensor framework is technically feasible and may provide health-relevant information that neither task delivers independently.

Nevertheless, several gaps remain. First, much of the existing literature has focused on benchmark datasets, older adults, or clinical and rehabilitation settings rather than the everyday activity ecology of university students (2, 10, 13). Widely used benchmark datasets such as UCI-HAR and PAMAP2 span broad age ranges and controlled laboratory conditions that may not adequately reflect the fragmented, campus-specific movement patterns of young adults in higher education. Second, many studies treat activity recognition as an end in itself, although its practical value increases when recognized behaviors can be mapped onto health-relevant constructs such as intensity categories or energy expenditure levels. Third, energy expenditure inference often depends on multi-sensor systems or tightly controlled laboratory protocols, which can limit feasibility in real-world educational settings (25, 26). Fourth, subject-independent validation through leave-one-subject-out cross-validation remains inconsistently applied, and studies relying on random window-level splitting may overestimate generalization performance in practice (26, 27).

Energy expenditure level classification offers an important bridge between engineering-oriented behavior recognition and public health interpretation. The Compendium of Physical Activities provides a standardized framework for assigning MET values to common activities (28). On this basis, sensor-detected activities can be mapped into low-, moderate-, and vigorous-intensity categories aligned with physical activity guidelines (1, 28). For university health monitoring, a system capable of identifying both daily behaviors and their intensity levels may be more informative for behavioral surveillance than a behavior classifier alone, as it directly relates sensor output to the intensity thresholds used in clinical and public health guidelines. Such a framework could support monitoring of sedentary time, ambulatory activity, and vigorous movement exposure in campus life.

Against this background, the present study developed and validated a waist-worn IMU-based framework for daily physical activity recognition and energy expenditure level classification in a university student cohort. The study addressed three questions. First, can a single waist-worn IMU accurately recognize representative daily activities in university students under subject-independent validation? Second, can the same inertial signals support classification of low-, moderate-, and vigorous-intensity energy expenditure levels? Third, how do alternative machine learning and deep learning models compare under leave-one-subject-out cross-validation? In addressing these questions, the present study fills three specific gaps that existing literature cannot close. First, it has not been established whether a single waist-worn IMU can achieve subject-independent generalization specifically for the fragmented, campus-typical movement ecology of university students—a population whose activity patterns differ structurally from the heterogeneous adult cohorts and controlled laboratory conditions underlying benchmark datasets such as UCI-HAR and PAMAP2; prior work cannot answer this question because it was not designed for this population or deployment context, with studies reporting strong stair discrimination predominantly using multi-placement or multi-sensor configurations (10, 17), or evaluating heterogeneous adult populations under window-level splitting (18, 22), none of which were designed to establish single-sensor waist-placement sufficiency for a homogeneous student cohort under strict LOSO cross-validation. Second, while activity recognition and energy expenditure estimation have been pursued in parallel research streams, no prior study has demonstrated that a single-sensor pipeline can simultaneously deliver both outputs in a form directly aligned with public health intensity guidelines, without requiring *post hoc* label mapping or additional instrumentation; the accuracy trade-off incurred by integrating these two tasks within a single-sensor framework had not previously been quantified for any student population (23, 24). Third, several high-accuracy CNN-LSTM and Inception-based studies in this domain rely on window-level random splitting rather than strict subject-independent validation; the performance estimates they report cannot be assumed to hold under LOSO conditions for a university student cohort, and this gap has not previously been verified at a sample scale of 96 participants (21, 22, 26, 27). By combining activity recognition with energy expenditure level classification, this study aims to contribute an application-oriented framework for wearable sensing research and campus physical activity monitoring.

## 2 Materials and methods

### 2.1 Study design and participants

**Figure 1:**
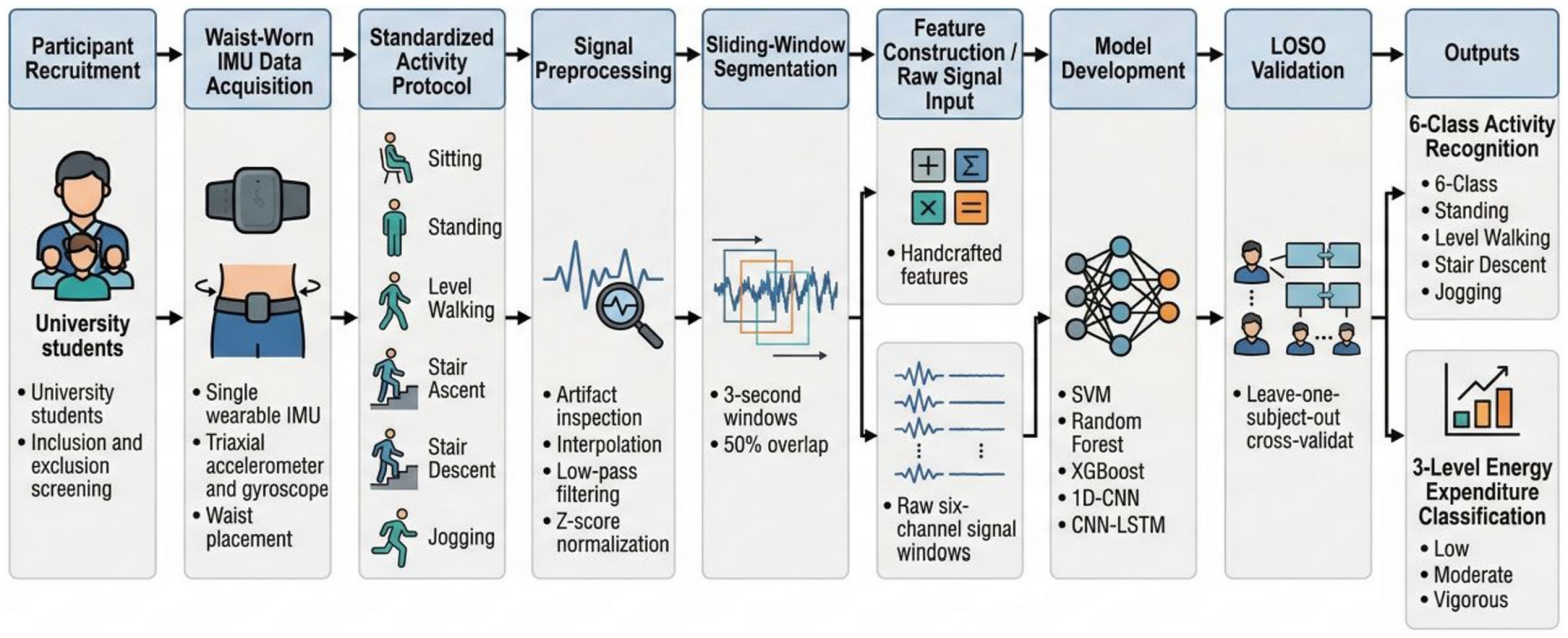
Overall study workflow, including participant recruitment, IMU-based data acquisition, preprocessing, feature construction, activity recognition, and energy expenditure level classification.

This study employed a sensor-based experimental design to develop and evaluate a wearable IMU framework for daily activity recognition and energy expenditure level classification. The analytical process comprised participant recruitment, standardized signal acquisition, preprocessing, sliding-window segmentation, feature construction, model development, and performance evaluation. Figure 1 summarizes the complete workflow from recruitment to classification output.

Participants were recruited from a comprehensive university through campus notices and class-based announcements. Inclusion criteria required participants to be full-time university students aged 18 to 25 years, capable of independently completing all activity tasks, and free from diagnosed neurological, musculoskeletal, cardiovascular, or vestibular disorders that could affect routine movement. Students were excluded if they had sustained recent lower-limb injuries, reported acute illness during testing, or generated incomplete recordings.

A total of 102 students volunteered. After screening and data quality inspection, 96 participants were retained in the final sample, including 48 women and 48 men. The final sample had a mean age of 20.4 ± 1.7 years, a mean height of 169.8 ± 8.1 cm, a mean body mass of 63.2 ± 10.6 kg, and a mean BMI of 21.8 ± 2.7 kg/m^2^. The demographic profile of the retained sample is presented in Table 1.

**Table 1:** Demographic characteristics of participants (*N* = 96).

Variable	Total sample	Male	Female
Age (years), mean ± SD	20.4 ± 1.7	20.6 ± 1.8	20.2 ± 1.6
Height (cm), mean ± SD	169.8 ± 8.1	175.6 ± 5.9	164.0 ± 5.7
Weight (kg), mean ± SD	63.2 ± 10.6	69.8 ± 9.1	56.6 ± 7.3
BMI (kg/m^2^), mean ± SD	21.8 ± 2.7	22.6 ± 2.5	21.0 ± 2.6
Undergraduate, *n* (%)	79 (82.3)	39 (81.3)	40 (83.3)
Postgraduate, *n* (%)	17 (17.7)	9 (18.7)	8 (16.7)
Regular exercise participation (≥3 times/week), *n* (%)	41 (42.7)	24 (50.0)	17 (35.4)

All participants received a written explanation of the study procedures and signed informed consent prior to data collection. They were instructed to wear comfortable sports shoes and non-restrictive daily clothing and to avoid vigorous exercise, alcohol, and excessive caffeine intake within 12 h before testing.

### 2.2 Wearable device and experimental protocol

**Figure 2:**
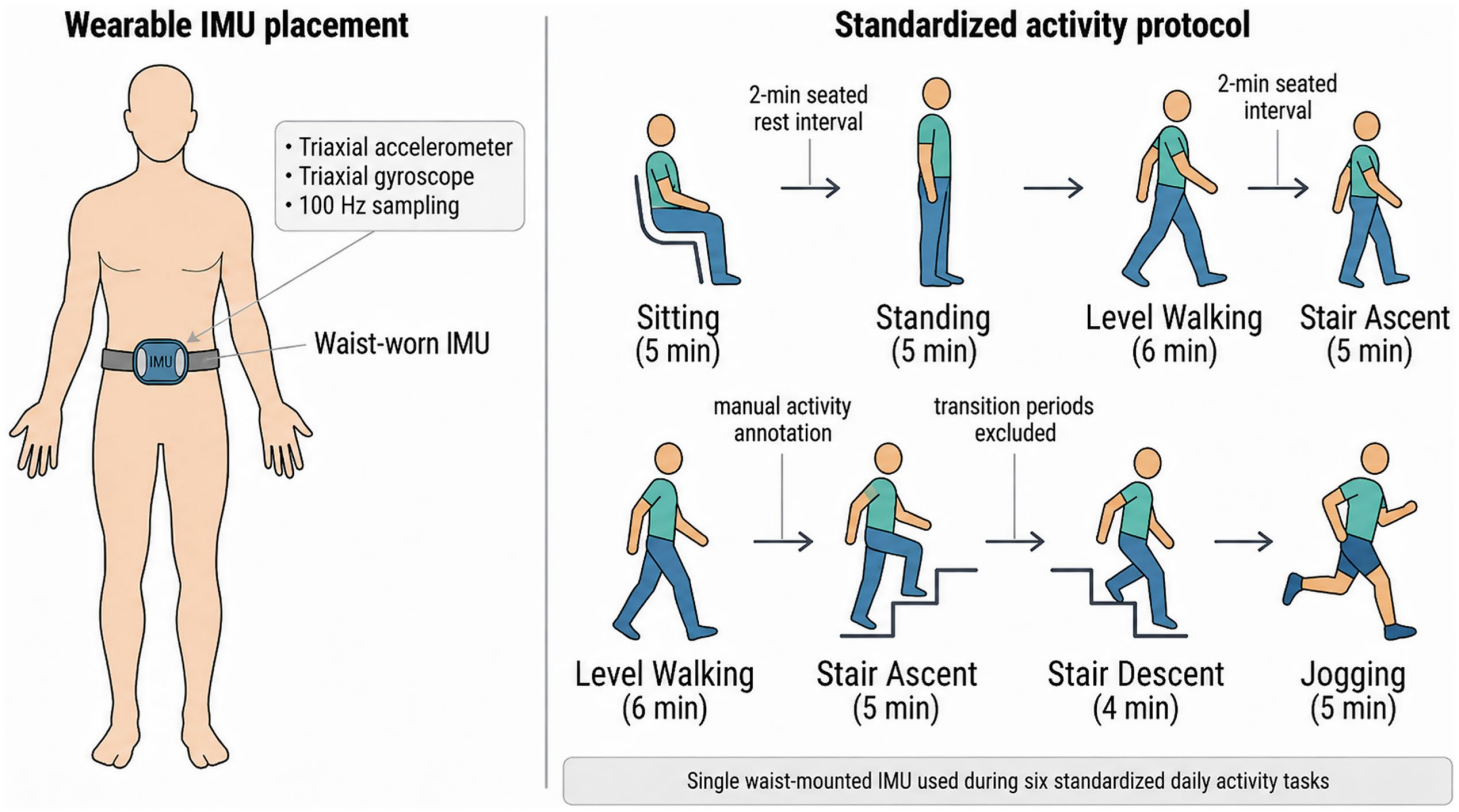
Wearable IMU placement at the anterior waist and the standardized activity protocol used for data acquisition.

A single waist-worn IMU was used to record triaxial acceleration and triaxial angular velocity signals. Waist placement was selected because it provides stable access to whole-body motion while remaining practical for potential real-world deployment (17). The device was fixed at the anterior waistline near the body’s approximate center of mass using an elastic belt. Figure 2 illustrates the device placement and standardized activity protocol.

The IMU comprised a triaxial accelerometer and a triaxial gyroscope. Signals were sampled at 100 Hz. The accelerometer range was set to ±8 g and the gyroscope range to ±500°/s. Data were transmitted to a mobile terminal and stored locally for backup. The device specifications and acquisition settings are summarized in Table 2.

**Table 2:** Specifications of the wearable IMU device and acquisition settings.

Item	Specification
Sensor type	Wearable inertial measurement unit
Channels	Triaxial accelerometer + triaxial gyroscope
Accelerometer range	±8 g
Gyroscope range	±500°/s
Sampling frequency	100 Hz
Resolution	16-bit
Placement	Anterior waist
Fixation method	Elastic belt
Recording environments	Indoor corridor, stairwell, campus jogging path

Six representative activity conditions were selected: sitting, standing, level walking, stair ascent, stair descent, and jogging. These activities were chosen because they reflect common movement patterns in university life while spanning low-, moderate-, and vigorous-intensity behaviors. Sitting and standing were each recorded for 5 min, level walking for 6 min, stair ascent for 5 min, stair descent for 4 min, and jogging for 5 min. A 2-min seated rest interval was inserted between adjacent tasks. All activity segments were manually annotated by trained researchers, and transition periods were excluded from the final labeled dataset.

The mapping between activity categories and representative MET values is shown in Table 3. Based on the Compendium of Physical Activities (28), sitting and standing were classified as low intensity, level walking and stair descent as moderate intensity, and stair ascent and jogging as vigorous intensity.

**Table 3:** Activity categories and corresponding energy expenditure levels.

Activity category	Duration	Representative MET value	Energy expenditure level
Sitting	5 min	1.3	Low
Standing	5 min	1.8	Low
Level walking	6 min	3.5	Moderate
Stair ascent	5 min	8.8	Vigorous
Stair descent	4 min	4.0	Moderate
Jogging	5 min	7.0	Vigorous

After excluding transition periods and corrupted segments, the total valid recording time was 2,659.4 min.

### 2.3 Signal processing and feature construction

All data were processed offline in Python. Raw signals were first inspected to identify missing samples and communication artifacts. Minor discontinuities were corrected by linear interpolation where appropriate. A fourth-order zero-phase Butterworth low-pass filter with a cutoff frequency of 20 Hz was applied to each channel to reduce high-frequency noise while preserving movement-related information (15, 29). Signals were then standardized using z-score normalization computed within the training data of each LOSO validation fold, so that no information from test participants contributed to normalization parameters.

**Figure 3:**
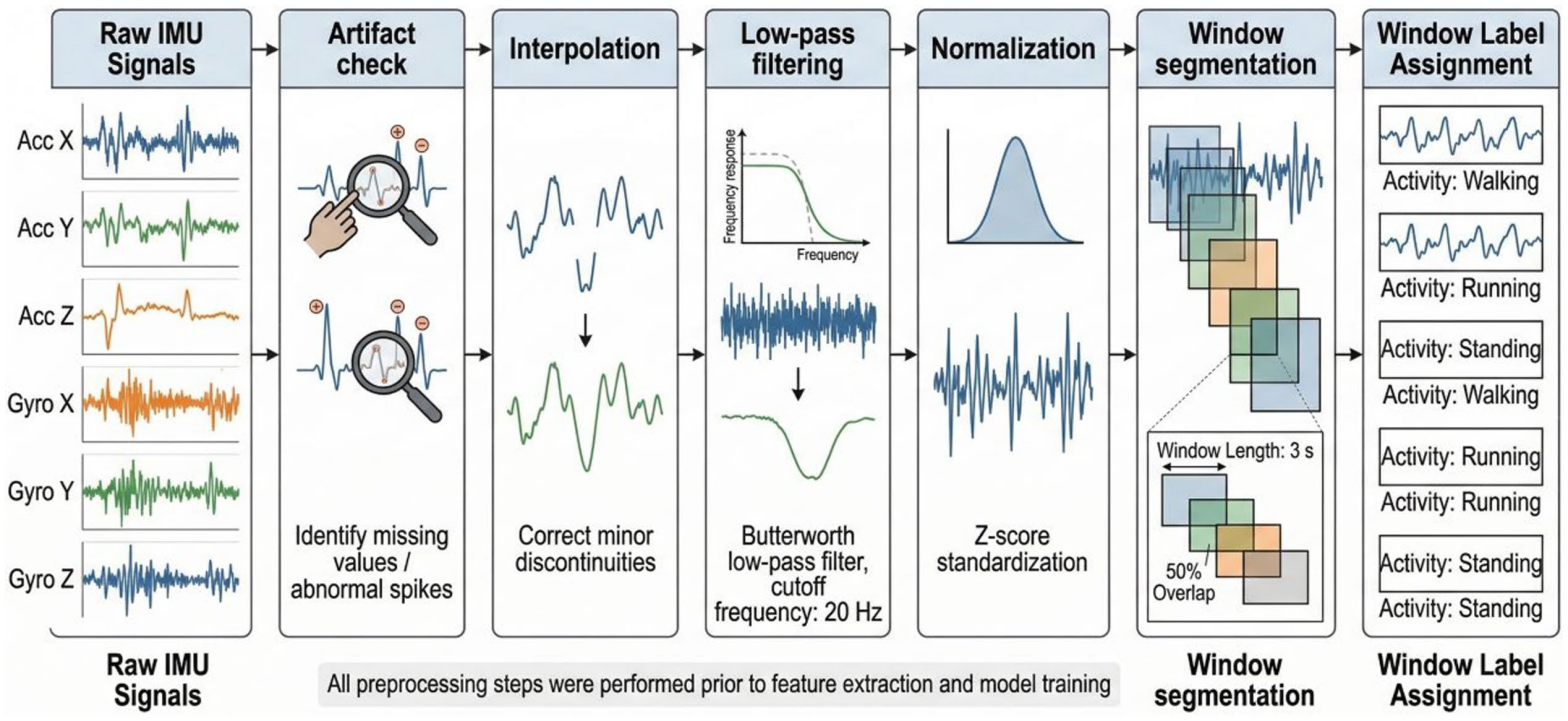
Signal preprocessing and segmentation procedure, including filtering, normalization, sliding-window segmentation, and label assignment.

The preprocessing and segmentation procedure is illustrated in Figure 3. After filtering and normalization, signals were segmented using a 3-s sliding window with 50% overlap. At a sampling rate of 100 Hz, each window contained 300 samples. This configuration was chosen to balance temporal stability and responsiveness and is consistent with established practice in HAR research (15, 17).

A total of 106,376 valid windows were retained after preprocessing. The distribution of windows by activity category is shown in Table 4.

**Table 4:** Number of valid signal windows by activity category.

Activity category	Valid duration (min)	Number of windows
Sitting	461.7	18,468
Standing	454.2	18,168
Level walking	543.8	21,752
Stair ascent	352.4	14,096
Stair descent	350.9	14,036
Jogging	496.4	19,856
Total	**2,659.4**	**106,376**

The bold values in this table represent the total sum of valid duration and number of windows.

For feature-based models, 96 hand-crafted features were extracted from each window. Features were computed across six raw signal channels (three accelerometer axes: a_x, a_y, a_z; three gyroscope axes: g_x, g_y, g_z) and two derived vector magnitude channels defined as VM_acc = √(a_x^2^ + a_y^2^ + a_z^2^) and VM_gyro = √(g_x^2^ + g_y^2^ + g_z^2^). For each channel, a windowed signal x = {x₁, x₂, …, x_N} of length N = 300 was summarized by the following six time-domain statistics: mean *μ* = (1/N)Σxᵢ, standard deviation *σ* = √[(1/N)Σ(xᵢ − μ)^2^], root mean square RMS = √[(1/N)Σxᵢ^2^], signal range R = max(x) − min(x), skewness S = (1/N)Σ[(xᵢ − μ)/σ]^3^, and kurtosis K = (1/N)Σ[(xᵢ − μ)/σ]^4^. Three frequency-domain descriptors were additionally extracted from the one-sided power spectral density P(k) = |X(k)|^2^/N, where X(k) = Σxᵢ·e^(−j2πki/N) is the discrete Fourier transform: dominant frequency f_dom = argmax_k P(k), spectral energy E = Σ_k P(k), and spectral entropy H = −Σ_k p̂(k)·log₂p̂(k) with p̂(k) = P(k)/Σ_k P(k). Spectral entropy is particularly informative for activity discrimination because it distinguishes periodic locomotion (low entropy, power concentrated in a narrow frequency band) from quasi-static postures (high entropy, diffuse power distribution). Applying these nine descriptors across eight channels yields the complete 72-dimensional feature vector per window. Features were computed independently within each LOSO training fold, and no statistics derived from test-fold data were used at any stage.

For deep learning models, the normalized six-channel windows were used directly as input without manual feature engineering.

### 2.4 Model development and evaluation

Two classification tasks were defined. The first was six-class daily physical activity recognition. The second was three-class energy expenditure level classification based on the low-, moderate-, and vigorous-intensity labels derived from Table 3.

**Figure 4:**
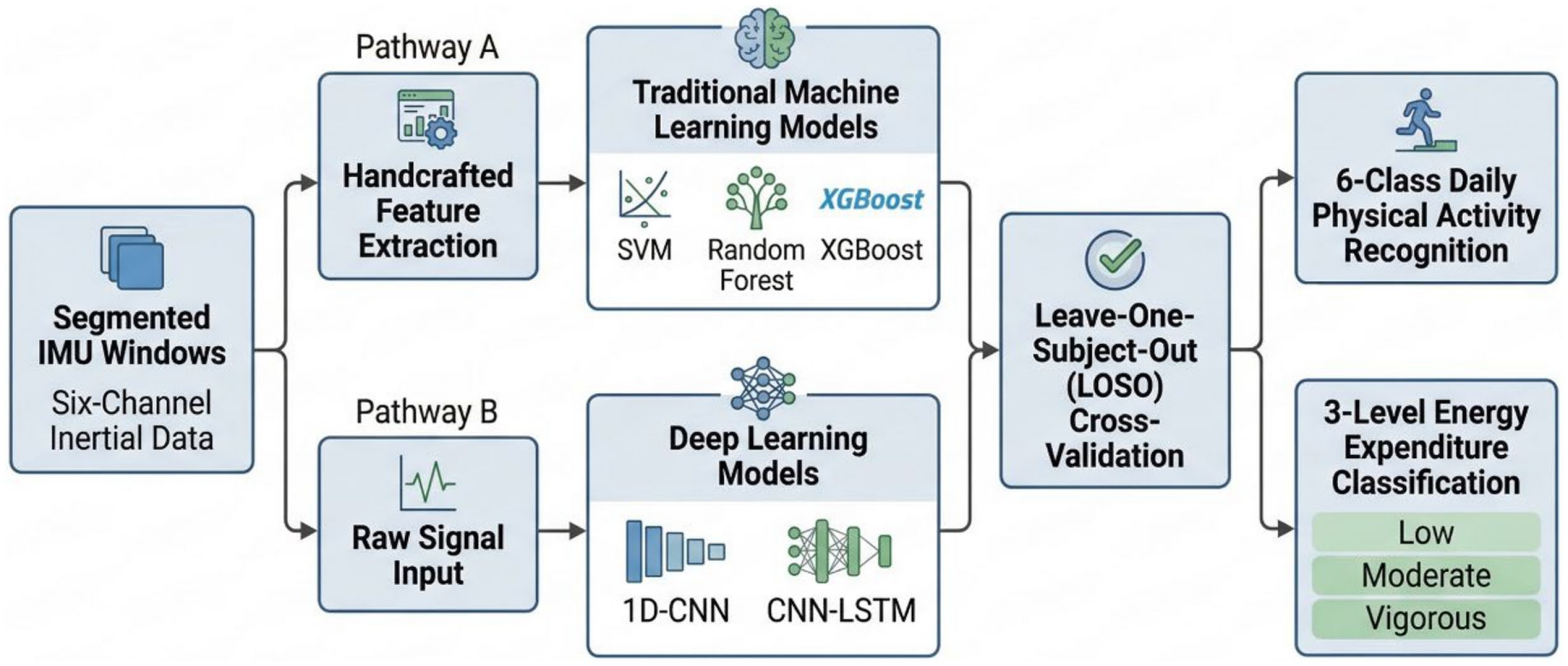
Model development pipeline for six-class activity recognition and three-class energy expenditure level classification.

Three traditional machine learning models were implemented: support vector machine (SVM) with a radial basis function kernel, random forest (RF) with 300 trees, and XGBoost (30). Two deep learning models were also evaluated: a one-dimensional convolutional neural network (1D-CNN) and a CNN-LSTM hybrid model. The 1D-CNN consisted of three convolutional blocks followed by global average pooling and a dense classifier. The CNN-LSTM model combined temporal convolution layers with an LSTM layer to capture both local features and sequential dependencies in the sensor stream (11, 19). These five models were selected to cover the spectrum from hand-crafted feature pipelines to end-to-end raw-signal representation learning, allowing a systematic comparison of modeling strategies under identical validation conditions (10, 11, 20, 30). Figure 4 outlines the classification framework.

The CNN-LSTM architecture accepted a raw input tensor of shape (300, 6) and processed it through three sequential one-dimensional convolutional blocks before passing the resulting feature sequence to a recurrent layer. The first convolutional block applied 64 filters of kernel size 3 with ReLU activation, batch normalization, and a dropout rate of 0.2 to extract low-level temporal features from the inertial signal; the second block increased the filter count to 128 to capture higher-order composite patterns; the third block reduced the representation back to 64 filters prior to sequential modeling. The output of the convolutional sub-network was passed to a single LSTM layer with 128 units, which maintained a cell state and hidden state across time steps to model long-range sequential dependencies in the sensor stream (11); a dropout rate of 0.3 was applied to the LSTM output. The final hidden state was forwarded to a fully connected layer of 64 units with ReLU activation, followed by a softmax output layer producing a probability distribution over C classes, where C = 6 for activity recognition and C = 3 for energy expenditure classification. The model was trained by minimizing categorical cross-entropy loss using the Adam optimizer with an initial learning rate of 10^−3^. Training was conducted in mini-batches of 64 windows, and early stopping with a patience of 10 epochs was applied based on held-out validation macro-F1 to prevent overfitting.

For the SVM model, the regularization parameter C and kernel coefficient *γ* were tuned by grid search within the training fold. For RF, tree depth and minimum samples per leaf were tuned; for XGBoost, learning rate, maximum tree depth, and the subsampling ratio were optimized. For the 1D-CNN and CNN-LSTM models, hyperparameters tuned within each LOSO training fold included the number of convolutional filters (candidates: {32, 64, 128}), the number of LSTM units (candidates: {64, 128, 256}), the dropout rate (candidates: {0.1, 0.2, 0.3, 0.4}), and the initial learning rate (candidates: {10^−2^, 10^−3^, 10^−4^}). All hyperparameter tuning was conducted exclusively on training data to prevent any form of data leakage.

Subject-independent generalization was assessed using leave-one-subject-out (LOSO) cross-validation. In each fold, data from one participant were reserved for testing, and all remaining participant data were used for training and hyperparameter tuning. This strategy prevents the leakage of participant-specific movement patterns into the test set and provides a more conservative and realistic estimate of real-world generalization than random window-level splitting (26, 27).

Performance was evaluated using accuracy, macro-precision, macro-recall, and macro-F1 score. Because both classification tasks involved multiple classes with minor imbalance across some folds, macro-F1 was used as the principal metric in model comparison. Confusion matrices were also generated to examine class-specific error patterns.

### 2.5 Statistical analysis

Continuous variables are reported as mean ± standard deviation and categorical variables as counts and percentages. To assess whether observed performance differences among models reflected systematic effects rather than fold-level variation, fold-level macro-F1 scores were compared across classifiers using the Friedman test, a non-parametric repeated-measures procedure that makes no distributional assumptions and is appropriate for comparing multiple models evaluated on the same set of LOSO folds (26). Pairwise *post hoc* comparisons were conducted using the Wilcoxon signed-rank test, with Bonferroni correction applied to control the familywise error rate across all pairwise combinations. Statistical significance was defined as *p* < 0.05.

The study protocol was reviewed and approved by the Beijing Sport University Exercise Science Laboratory Ethics Committee. All procedures were conducted in accordance with the Declaration of Helsinki. Participants were informed that participation was voluntary and that they could withdraw at any time without penalty. Written informed consent was obtained from all participants prior to data collection.

## 3 Results

### 3.1 Dataset characteristics

The final dataset comprised 106,376 valid signal windows derived from 2,659.4 min of analyzed IMU recordings after the exclusion of transition periods and corrupted segments. As summarized in Table 4, the six activity categories were represented with acceptable balance across the dataset, although walking and jogging contributed somewhat more windows than stair-related tasks. Walking yielded the largest number of valid windows (*n* = 21,752), followed by jogging (*n* = 19,856), sitting (*n* = 18,468), standing (*n* = 18,168), stair ascent (*n* = 14,096), and stair descent (*n* = 14,036).

**Figure 5:**
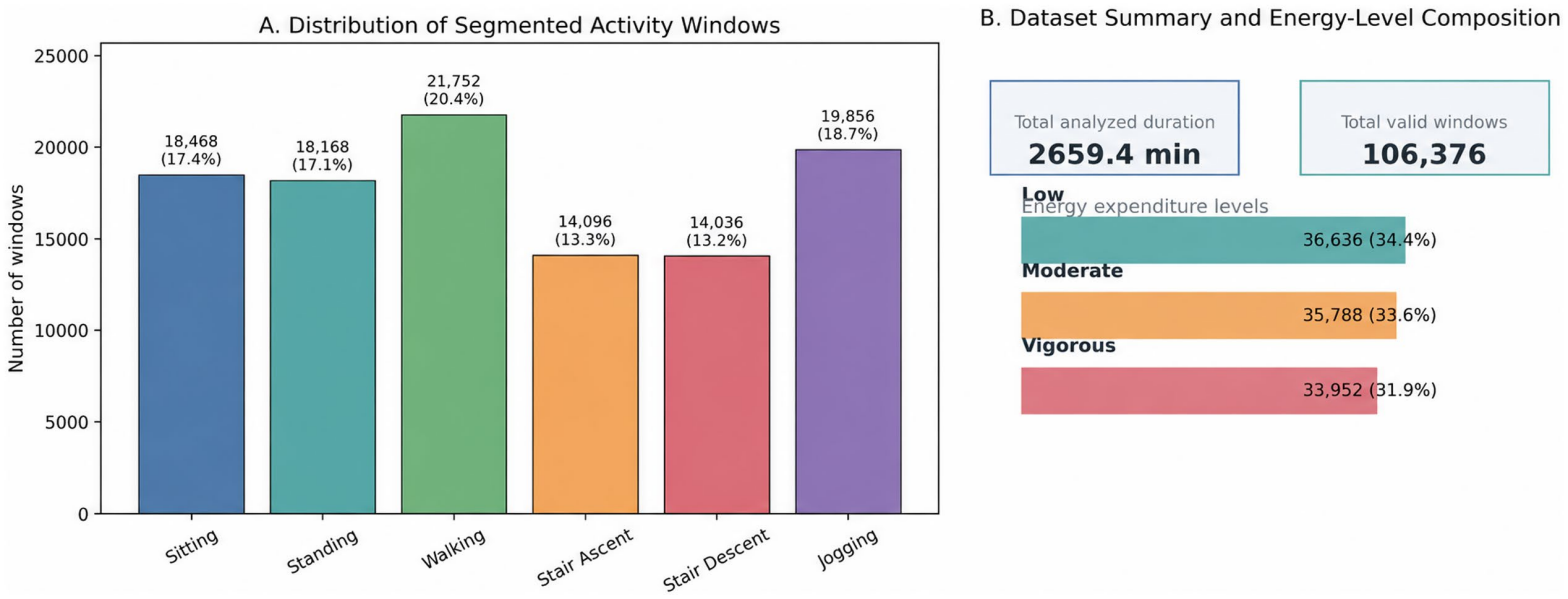
Overview of the analyzed dataset.

To provide a clearer overview of class composition, Figure 5 presents the distribution of segmented activity windows as well as the overall dataset summary. As shown in Figure 5A, no single activity class overwhelmingly dominated the full dataset, which reduced the risk that model performance would be driven primarily by one high-frequency class. Figure 5B further summarizes the total analyzed duration and total number of valid windows, and shows the composition of the three energy expenditure levels derived from the six activity classes. When the activity windows were mapped to energy expenditure levels, low-intensity windows accounted for 36,636 samples (34.4%), moderate-intensity windows accounted for 35,788 samples (33.6%), and vigorous-intensity windows accounted for 33,952 samples (31.9%). The class distribution remained sufficiently balanced for both six-class activity recognition and three-class energy expenditure classification tasks.

The dataset characteristics are broadly consistent with those reported in prior wearable HAR studies of comparable scope. A total of 106,376 windows derived from 96 participants under LOSO validation provides a reasonable empirical basis for subject-independent model evaluation, as studies of similar design and participant scale have reported stable performance estimates from datasets of this order of magnitude (15, 17, 27). The near-equal distribution across intensity levels additionally supports the interpretability of the subsequent model comparisons, given that macro-F1 scores are less sensitive to class-frequency artifacts when class sizes are approximately balanced.

### 3.2 Performance of daily physical activity recognition

**Figure 6:**
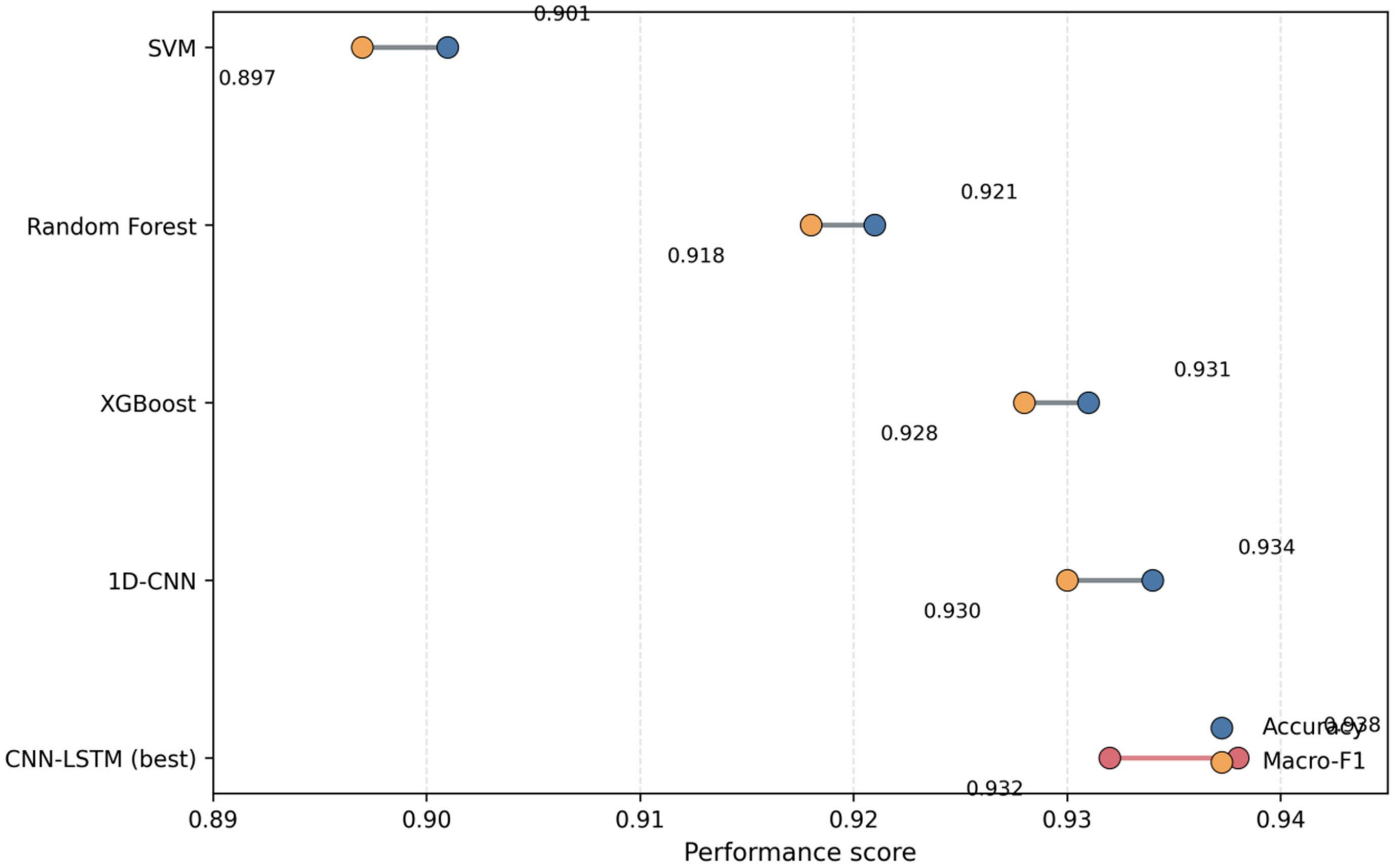
Model comparison for six-class daily physical activity recognition.

The first target task was six-class daily physical activity recognition. Under leave-one-subject-out validation, all five candidate models achieved strong overall performance, with the deep learning models consistently outperforming the traditional machine learning approaches. The full model comparison is reported in Table 5, and the corresponding visual summary is shown in Figure 6.

**Table 5:** Overall performance of different models for six-class daily physical activity recognition under LOSO validation.

Model	Accuracy	Macro-precision	Macro-recall	Macro-F1
SVM	0.901	0.898	0.896	0.897
Random forest	0.921	0.919	0.917	0.918
XGBoost	0.931	0.928	0.927	0.928
1D-CNN	0.934	0.930	0.929	0.930
CNN-LSTM	**0.938**	**0.933**	**0.931**	**0.932**

The bold values in this table indicate the best-performing model (highest scores) across the respective evaluation metrics.

As shown in Table 5 and Figure 6, the CNN-LSTM model achieved the best overall performance, with an accuracy of 0.938 and a macro-F1 score of 0.932. The 1D-CNN model followed closely, with an accuracy of 0.934 and a macro-F1 score of 0.930. Among the traditional machine learning models, XGBoost performed best, reaching an accuracy of 0.931 and a macro-F1 score of 0.928, whereas Random Forest and SVM showed comparatively lower performance. Although the absolute gap between the top deep learning model and the strongest feature-based model was modest, the ranking pattern was consistent across both evaluation metrics.

To examine class-level performance in greater detail, the precision, recall, and F1-score of the best-performing CNN-LSTM model are reported in Table 6. Sitting and jogging were recognized most accurately, with F1-scores of 0.970 and 0.945, respectively. Standing and level walking also showed strong discrimination, with F1-scores above 0.92. By contrast, stair descent had the lowest class-wise F1-score (0.896), followed by stair ascent (0.912), indicating that stair-related activities were more difficult to separate than sedentary and highly dynamic locomotor behaviors.

**Table 6:** Class-wise performance of the CNN-LSTM model for daily physical activity recognition.

Activity category	Precision	Recall	F1-score
Sitting	0.968	0.973	0.970
Standing	0.944	0.938	0.941
Level walking	0.926	0.931	0.928
Stair ascent	0.918	0.907	0.912
Stair descent	0.901	0.892	0.896
Jogging	0.943	0.947	0.945

**Figure 7:**
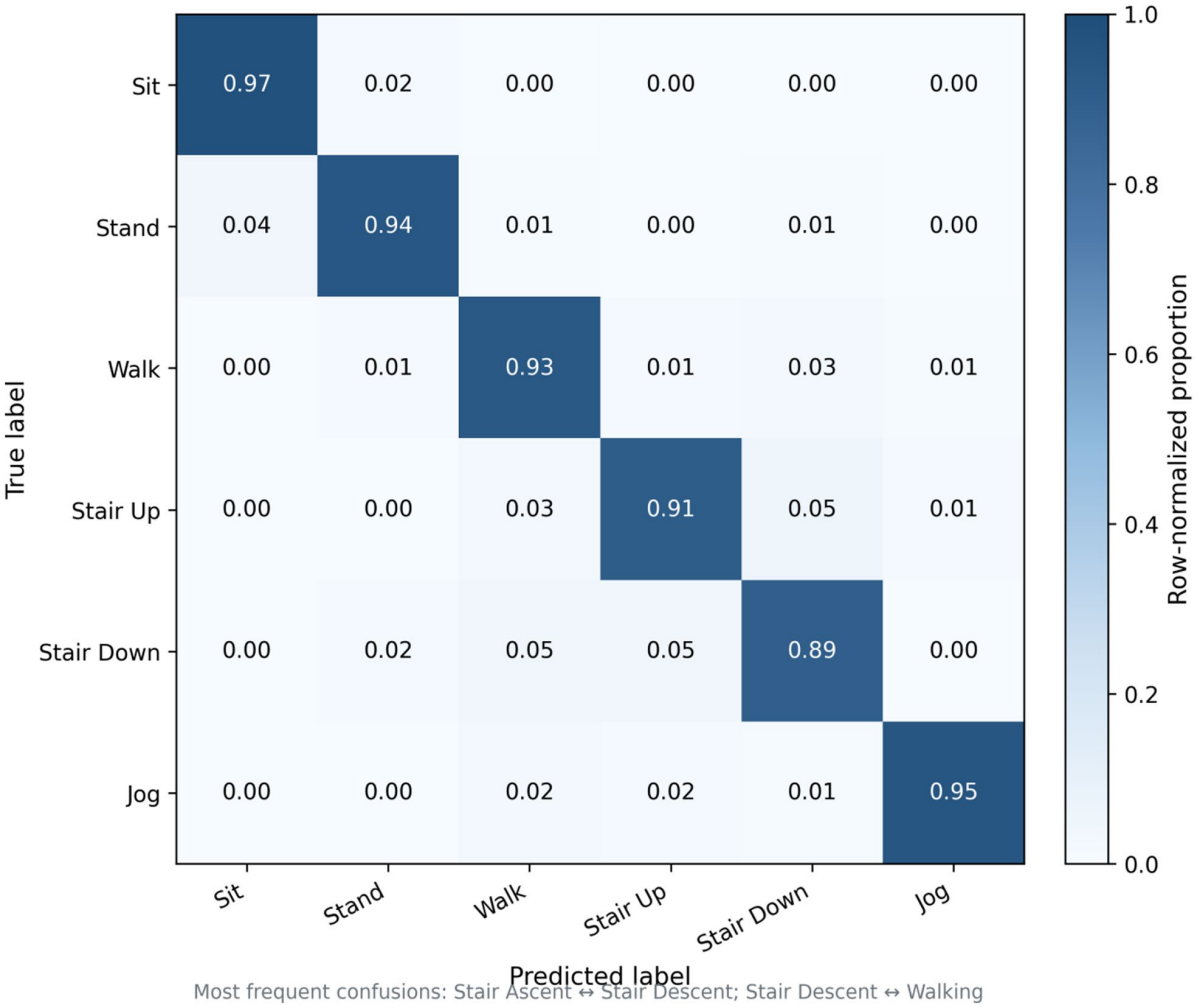
Confusion matrix for six-class activity recognition.

This error structure is illustrated in Figure 7, which presents the row-normalized confusion matrix for the CNN-LSTM model.

As shown in Figure 7, the most frequent misclassifications occurred between stair ascent and stair descent, and between stair descent and walking. Confusion between sitting and standing was limited, and confusion between sedentary and vigorous activities was rare. This pattern indicates that the classifier preserved broad behavioral distinctions effectively, while most errors occurred between movement classes sharing similar rhythmic and postural characteristics.

### 3.3 Performance of energy expenditure level classification

**Figure 8:**
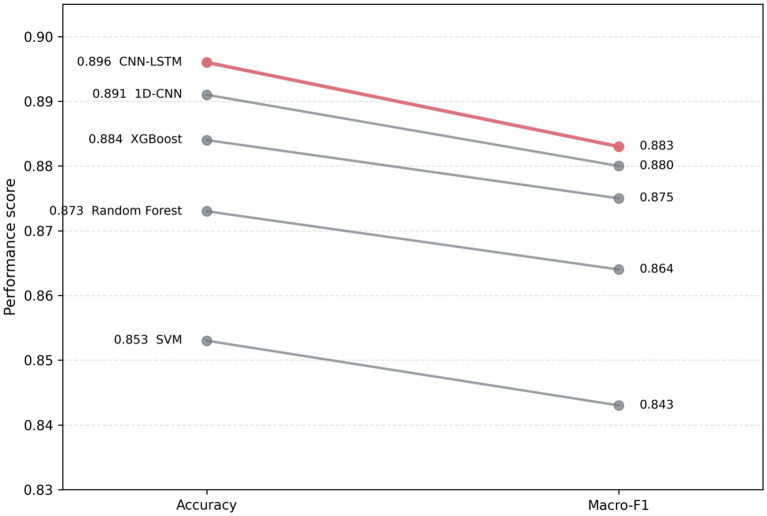
Model comparison for energy expenditure level classification.

The second target task involved classifying segmented windows into low-, moderate-, and vigorous-intensity energy expenditure levels. Overall classification performance was somewhat lower than that observed for six-class activity recognition, but remained strong across all models. The full comparison is shown in Table 7, and the corresponding visualization is provided in Figure 8.

**Table 7:** Overall performance of different models for three-class energy expenditure level classification under LOSO validation.

Model	Accuracy	Macro-precision	Macro-recall	Macro-F1
SVM	0.853	0.845	0.842	0.843
Random Forest	0.873	0.866	0.863	0.864
XGBoost	0.884	0.877	0.874	0.875
1D-CNN	0.891	0.881	0.879	0.880
CNN-LSTM	**0.896**	**0.885**	**0.881**	**0.883**

The bold values in this table indicate the best-performing model (highest scores) across the respective evaluation metrics.

As reported in Table 7 and Figure 8, the CNN-LSTM model again achieved the best overall performance, with an accuracy of 0.896 and a macro-F1 score of 0.883. The 1D-CNN model ranked second, reaching an accuracy of 0.891 and a macro-F1 score of 0.880. XGBoost remained the strongest traditional machine learning model, with an accuracy of 0.884 and a macro-F1 score of 0.875. Random Forest and SVM showed lower performance, but both still produced acceptable classification results.

Class-wise performance of the best-performing CNN-LSTM model is summarized in Table 8. Low-intensity activity was classified most accurately, with a precision of 0.943, a recall of 0.951, and an F1-score of 0.947. Moderate-intensity activity yielded a recall of 0.841 and an F1-score of 0.849, whereas vigorous-intensity activity yielded a recall of 0.851 and an F1-score of 0.853. These results indicate that the separation of low-intensity behavior from higher-intensity behavior was comparatively strong, whereas the distinction between adjacent moderate- and vigorous-intensity categories was more challenging.

**Table 8:** Class-wise performance of the CNN-LSTM model for energy expenditure level classification.

Energy expenditure level	Precision	Recall	F1-score
Low	0.943	0.951	0.947
Moderate	0.857	0.841	0.849
Vigorous	0.856	0.851	0.853

**Figure 9:**
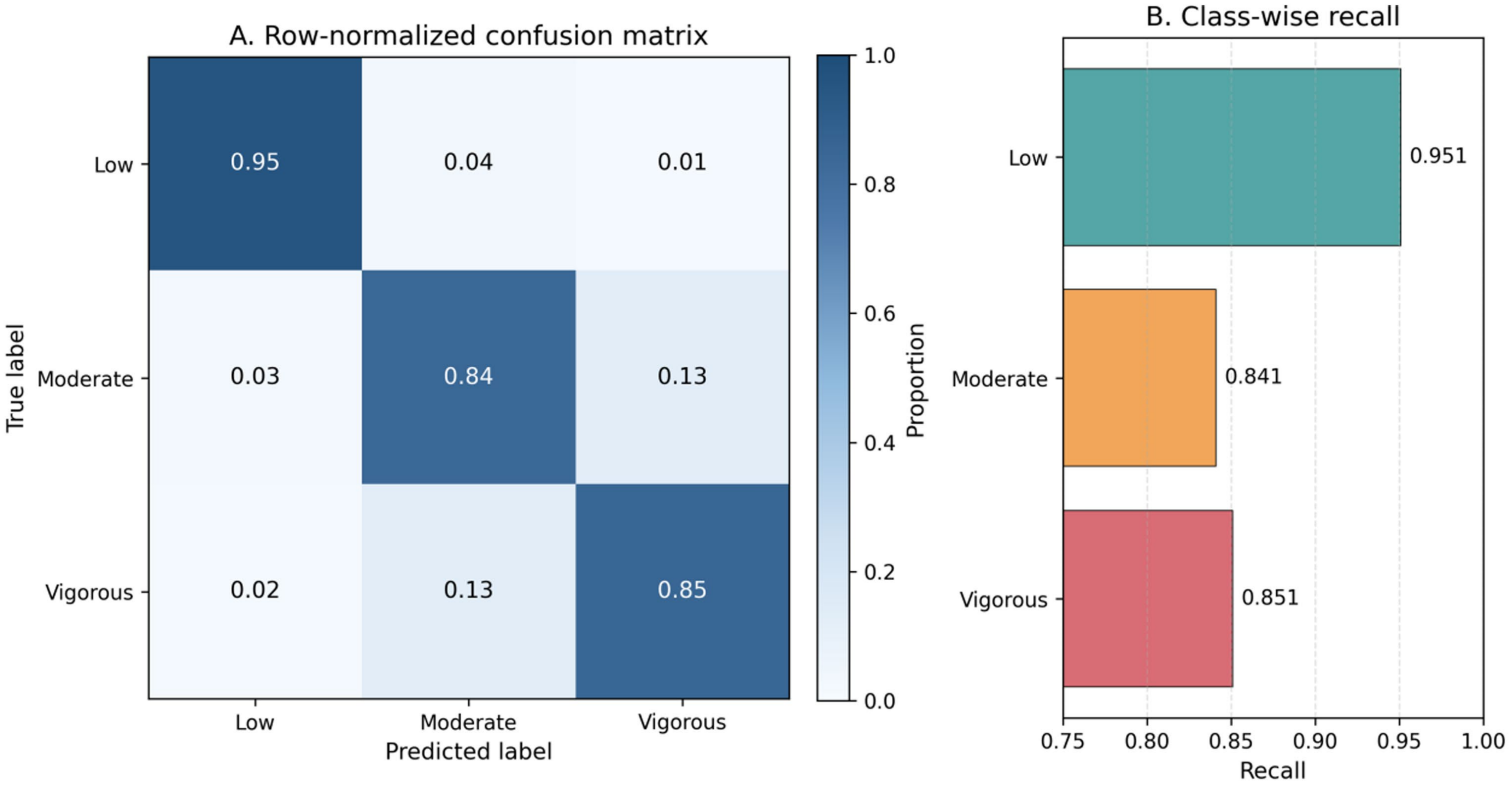
Performance profile for three-class energy expenditure classification.

Figure 9 presents the row-normalized confusion matrix together with the class-wise recall profile for the CNN-LSTM model.

As shown in Figure 9A, the principal classification ambiguity occurred between moderate and vigorous energy expenditure levels. Moderate-intensity windows were misclassified as vigorous-intensity windows more frequently than as low-intensity windows, and vigorous-intensity windows showed a similar tendency to be confused with moderate intensity rather than with low intensity. Figure 9B confirms this pattern, showing the highest recall for the low-intensity class (0.951), followed by vigorous intensity (0.851) and moderate intensity (0.841). These results suggest that the proposed IMU-based framework can classify energy expenditure levels with reasonable accuracy, although classification stability was lower for adjacent moderate and vigorous intensity bands than for the low-intensity class.

### 3.4 Statistical comparison of model performance

To assess whether the observed performance differences among models reflected systematic effects rather than fold-level variation, fold-level macro-F1 scores were compared across classifiers using the Friedman test, a non-parametric repeated-measures procedure appropriate for comparing multiple models evaluated on the same set of LOSO folds. Pairwise *post hoc* comparisons were conducted using the Wilcoxon signed-rank test, with Bonferroni correction applied to control the familywise error rate.

For six-class daily physical activity recognition, the Friedman test indicated a statistically significant overall model effect (*χ*^2^(4) = 68.3, *p* < 0.001). *Post hoc* pairwise comparisons showed that the CNN-LSTM model significantly outperformed both SVM and Random Forest (*p* < 0.05 after Bonferroni correction). The difference between CNN-LSTM and XGBoost approached but did not reach statistical significance after correction (*p* = 0.08), which is consistent with the modest absolute gap between the two models observed in Table 5. The difference between CNN-LSTM and 1D-CNN was not statistically significant (*p* = 0.31), indicating that both deep learning architectures performed similarly on this task.

A similar pattern was observed for three-class energy expenditure classification. The Friedman test again indicated a significant overall effect (*χ*^2^(4) = 61.7, *p* < 0.001). Post hoc comparisons confirmed that the two deep learning models significantly outperformed SVM and Random Forest (*p* < 0.05 after correction). The difference between CNN-LSTM and XGBoost did not reach statistical significance after Bonferroni correction (*p* = 0.12), suggesting that XGBoost represented a competitive alternative among the models evaluated. The difference between CNN-LSTM and 1D-CNN was similarly non-significant (*p* = 0.27).

Taken together, the statistical analyses support two conclusions. First, the performance advantage of the best-performing models over the weaker baselines was not attributable to random fold-level variation. Second, the performance difference between CNN-LSTM and XGBoost was modest and did not reach statistical significance after correction, indicating that well-designed feature-based pipelines remain competitive with deep learning under subject-independent validation in this task context.

### 3.5 Summary of Main findings

The results of this study can be organized around three principal observations. First, a single waist-worn IMU enabled accurate recognition of six representative daily activities in university students, with the CNN-LSTM model achieving 93.8% accuracy and a macro-F1 score of 0.932 under LOSO validation. Second, the same inertial signals supported classification of low-, moderate-, and vigorous-intensity energy expenditure levels, with the CNN-LSTM model achieving 89.6% accuracy and a macro-F1 score of 0.883. Third, classification errors followed biomechanically interpretable patterns, occurring primarily between stair-related activities and between adjacent moderate- and vigorous-intensity categories, rather than between structurally distinct behavioral classes such as sedentary behaviors and vigorous locomotion.

## 4 Discussion

This study developed and validated a wearable IMU-based framework for daily physical activity recognition and energy expenditure level classification in university students. The results demonstrate that a single waist-worn IMU can support accurate recognition of six representative daily activities and can further classify movement into low-, moderate-, and vigorous-intensity categories. Under LOSO validation, the best-performing CNN-LSTM model achieved 93.8% accuracy for activity recognition and 89.6% accuracy for energy expenditure classification. These findings are discussed below in relation to prior work, the interpretability of the error patterns, the practical implications for campus health monitoring, the specific contributions of the present framework, and the limitations that should be considered in interpreting the results.

### 4.1 Activity recognition performance in relation to prior work

The activity recognition results are broadly consistent with earlier wearable HAR studies reporting strong performance from body-worn inertial sensors (9, 10, 15–17). In the present study, sitting and jogging were recognized most accurately, with F1-scores of 0.970 and 0.945, respectively. This is biomechanically plausible: sitting is characterized by stable low-amplitude trunk motion, whereas jogging produces clear periodic oscillation and larger dynamic range in all sensor channels. By contrast, the main classification difficulty was concentrated among stair ascent, stair descent, and level walking. These activities share cyclic lower-limb movement and partially overlapping trunk-level rhythmic patterns, which makes them harder to discriminate when only a single waist-mounted sensor is used. Similar confusion patterns have been documented in previous wearable HAR research, particularly under subject-independent validation (10, 15, 17). Recent work examining placement effects across body locations confirms that waist and trunk sensors provide stable recognition performance for locomotion-type activities but show greater ambiguity for transitional movements such as stair negotiation compared with ankle-mounted configurations (18).

To situate the present findings more precisely within the current literature, Table 9 provides a structured comparison of the proposed framework with representative wearable HAR studies using inertial sensors under subject-independent or cross-subject validation. The comparison now includes Inception-ResNet architectures (21) and sensor-placement studies (18) to reflect the broader range of modeling and hardware approaches in the current literature.

**Table 9:** Comparison of the proposed framework with representative wearable HAR studies.

Study	Sensor placement	Population	No. of activities	Best model	Validation	Best accuracy
Attal et al. (10)	Wrist, thigh, chest	Mixed adults	8	SVM	Cross-subject	0.870
Cleland et al. (17)	Wrist, hip, ankle	Mixed adults	7	kNN	Cross-subject	0.880
Khatun et al. (22)	Pocket (smartphone)	Mixed adults	4–6	CNN-LSTM	Cross-subject	0.931
Mekruksavanich and Jitpattanakul (21)	Wrist	Mixed adults	6	iSPLInception	Cross-subject	0.952
IMU placement study (18)	Wrist to ankle (multiple)	Mixed adults	6	CNN	Cross-subject	~0.91 (waist)
Zhang et al. (review) (19)	Various	Various	Various	Various	Various	Up to 0.96+
Present study	Waist (single IMU)	University students	6	CNN-LSTM	LOSO (*n* = 96)	0.938

Several observations emerge from this comparison. First, the present framework achieves accuracy comparable to or above that of multi-sensor studies from the earlier literature (10, 17), while using only a single sensor at the waist, which is a more practical configuration for large-scale monitoring. Second, the accuracy obtained here (93.8%) is consistent with the upper range reported for CNN-LSTM models in recent benchmark evaluations (19, 22), and remains competitive with Inception-ResNet architectures (21) despite not employing multi-scale convolutional design. Third, placement-focused studies confirm that waist-level sensors yield recognition accuracy in the range of approximately 91% for six-class tasks (18), providing external validation that the present waist-placement choice is appropriate for the target activity set. Fourth, the existing studies that report high accuracies are predominantly evaluated on heterogeneous adult populations or on public benchmark datasets such as UCI-HAR, rather than on the specific daily movement ecology of university students. The present study addresses this gap by collecting purpose-built data from a homogeneous student cohort under a protocol reflecting campus-typical activities, including stair use and jogging on a campus path, providing a more ecologically relevant validation of the framework for this target population.

### 4.2 Energy expenditure level classification

A distinguishing aspect of the present study is that it did not treat activity recognition as a terminal objective. Rather than stopping at behavior-level classification, detected activities were mapped into MET-informed energy expenditure levels. This matters because physical activity recommendations are formulated in terms of intensity, not merely activity labels (1). From a practical standpoint, knowing whether a student’s movement profile is dominated by low-, moderate-, or vigorous-intensity behavior is more directly actionable for health monitoring than a list of isolated activity labels.

The present results indicate that inertial data alone can support this form of classification with reasonable accuracy. Low-intensity activity was classified most accurately (F1 = 0.947), while the main ambiguity occurred between moderate and vigorous categories (F1-scores of 0.849 and 0.853, respectively). This pattern is understandable: moderate and vigorous movements may generate partially overlapping waist-motion profiles depending on individual walking pace, body morphology, and execution style, whereas low-intensity behavior such as sitting and standing produces distinctly low-amplitude signals that are easier to separate from higher-intensity classes (25, 26, 28, 31). The energy expenditure classification accuracy of 89.6% obtained here is broadly consistent with that reported in IMU-based energy expenditure estimation studies using similar sensor configurations and deep learning approaches (23, 24), though direct comparison is limited by differences in task definition, reference method, and population.

### 4.3 Model comparison

The model comparison results provide additional insight into the relative merits of deep learning and feature-based approaches in this context. Deep learning models performed slightly but consistently better than traditional machine learning models across both tasks, with CNN-LSTM showing the best point estimates overall. The performance advantage of CNN-LSTM over 1D-CNN was numerically small and not statistically significant under Bonferroni correction, suggesting that the additional sequential modeling capacity of the LSTM layer provided only marginal benefit over convolutional feature extraction alone for these activity classes. This likely reflects the fact that the 3-s window already captures sufficient temporal context for most of the target activities at 100 Hz.

The performance gap between CNN-LSTM and XGBoost was also modest and did not reach statistical significance after correction (*p* = 0.08 for activity recognition; *p* = 0.12 for energy expenditure classification). This finding suggests that well-designed hand-crafted features still encode a substantial portion of the discriminative information in waist-worn IMU signals, and that the choice between deep learning and feature-based approaches may depend less on predictive performance alone and more on operational factors such as computational cost, interpretability requirements, and deployment constraints. In applied settings such as campus wearable systems, where model transparency and edge-device compatibility may be priorities, XGBoost represents a practically relevant alternative to deep learning.

### 4.4 Implications for campus health monitoring

The present framework has several characteristics that may be relevant to practical deployment in university health monitoring contexts. Student daily behavior often includes long periods of sitting, fragmented walking, frequent stair use, and irregular bouts of exercise—a movement ecology that differs from the laboratory scenarios underlying many benchmark HAR datasets. A wearable IMU system designed and validated on this population could provide more objective information about behavior structure than questionnaire-based approaches (6, 7), particularly for short-duration behaviors such as stair use that are poorly captured by self-report.

The integration of MET-informed intensity classification within the same single-sensor framework represents a specific contribution of the present study relative to systems that perform activity recognition alone. By mapping recognized activities onto intensity categories directly aligned with public health guidelines (1, 28), the framework produces output that is interpretable within an established health communication framework, without requiring additional instrumentation or offline processing. This could facilitate the development of campus wellness programs, digital physical education tools, and individualized feedback systems that identify insufficient moderate-to-vigorous activity exposure or excessive sedentary time at the individual level.

To articulate the contribution of the present study with greater precision, it is useful to consider what a researcher in this field could not have known or concluded in the absence of this work. Three specific knowledge gaps existed that prior literature was not designed to close.

First, prior to this study, it was not known whether a single waist-worn IMU—without supplementary wrist, ankle, or chest sensors—is sufficient to achieve subject-independent discrimination of stair ascent, stair descent, and level walking within a homogeneous young adult student cohort. Studies reporting the strongest stair discrimination in the existing literature predominantly use multi-placement or multi-sensor configurations (10, 17), or validate on heterogeneous adult populations using window-level random splitting (22). Placement-focused studies confirm that waist-level performance for stair-related activities is lower than for ankle-mounted configurations (18), and Inception-ResNet architectures trained on wrist-worn sensors have been validated only on general adult populations (21)—neither body of work was designed for, or validated on, the specific campus-typical movement ecology of university students under strict LOSO cross-validation. A system designer could not have concluded from existing evidence alone that single-sensor waist placement achieves acceptable subject-independent stair discrimination for this target context; the present results—stair ascent F1 = 0.912 and stair descent F1 = 0.896 under LOSO across 96 participants—provide the first empirical basis for that conclusion.

Second, prior to this study, the accuracy trade-off incurred by integrating activity recognition and MET-informed energy expenditure level classification within a single-sensor pipeline had not been quantified for a university student population. Activity recognition and energy expenditure estimation have been pursued as largely separate research streams (19, 23, 24). No prior study had simultaneously validated both outputs from a single waist-worn IMU under subject-independent conditions in this population, nor had it been established whether the intensity classification output from such a pipeline reaches a level of accuracy that is practically useful for health surveillance. A researcher could not have predicted the performance gap between the two tasks (93.8% vs. 89.6%), nor known whether both outputs remain practically meaningful within a single-sensor framework, without the present evidence. The present study is the first to quantify this trade-off and to demonstrate that both outputs are sufficiently accurate to support campus health monitoring applications.

Third, prior to this study, it had not been established whether the high accuracies reported for CNN-LSTM and Inception-based models in the recent HAR literature—including results exceeding 93% (22) and 95% (21)—hold under strict LOSO validation specifically for a university student cohort performing campus-typical activities. Several studies achieving these accuracies rely on window-level random splitting (21, 22), which is known to inflate generalization estimates by allowing windows from the same participant to appear in both training and test sets (26, 27). A researcher could not have extrapolated from existing benchmark studies to conclude that comparable accuracy holds under true LOSO conditions for this specific deployment context; the present study confirms that it does (93.8%), but this confirmation required a purpose-built dataset and strict LOSO design that did not exist prior to this work.

Taken together, these three contributions represent empirical knowledge that was not available before this study: the sufficiency of single waist-sensor placement for stair discrimination under LOSO in a student cohort, the joint pipeline accuracy trade-off for recognition and intensity classification in this population, and a LOSO-validated performance benchmark for campus-typical activities at a 96-participant scale. Table 10 summarizes these gaps, the state of prior literature for each, and the specific evidence the present study contributes.

**Table 10:** Knowledge gaps that prior literature could not close and how the present study addresses them.

Knowledge gap	State of prior literature	What the present study establishes
Single waist-sensor sufficiency for stair discrimination in university students under LOSO	Strong stair discrimination reported only for multi-sensor/multi-placement systems (10), (17), heterogeneous adult populations with window-level splitting (22), or wrist-sensor Inception models (21); placement studies show waist accuracy lower than ankle for stair tasks (18); no evidence for single waist-sensor in student cohort under LOSO	First LOSO-validated evidence: stair ascent F1 = 0.912, stair descent F1 = 0.896 from single waist-IMU in 96 university students under campus-typical conditions
Accuracy trade-off of integrating recognition and intensity classification in a single-sensor pipeline for students	Recognition and energy expenditure estimation pursued as separate streams (19, 23, 24); no prior study simultaneously validated both outputs from single waist-sensor under LOSO in student population	First quantification of performance gap (93.8% vs. 89.6%); establishes that both outputs are sufficiently accurate for campus health surveillance within one pipeline
LOSO-validated performance benchmark for campus-typical activities at sufficient scale	High CNN-LSTM accuracies [>93% (22)] and Inception accuracies [>95% (21)] in recent literature predominantly based on window-level splitting; LOSO performance for student cohort at this scale not established (26), (27)	Purpose-built LOSO-validated benchmark (*n* = 96) confirming high accuracy holds under subject-independent conditions for campus-typical activities including stair use

Furthermore, the use of a single waist-worn sensor, as opposed to multi-sensor systems that place devices at the wrist, ankle, and chest simultaneously, substantially reduces the burden of data collection and the complexity of sensor synchronization. The present results suggest that this simpler configuration is sufficient for the recognition and intensity classification tasks examined here, at least within the activity set studied.

### 4.5 Limitations

Several limitations should be considered when interpreting the present findings.

First, the activity set was intentionally restricted to six representative behaviors and does not capture the full diversity of free-living student movement. Activities such as cycling, resistance training, standing desk work, or prolonged computer use were not included. The recognition and classification performance reported here therefore reflects performance under a constrained protocol rather than in naturalistic free-living conditions. Future work should extend the activity taxonomy and evaluate the framework during longer-term, unstructured monitoring periods to assess whether the performance characteristics observed here generalize to a broader behavioral repertoire.

Second, energy expenditure labels were assigned using representative MET values from the Compendium of Physical Activities (28) rather than individual physiological measurement. This approach is standard in large-scale wearable research and enables consistent classification across participants, but it does not account for inter-individual variability in metabolic cost. The actual energy expenditure for a given activity can differ meaningfully across individuals depending on body composition, cardiorespiratory fitness, movement efficiency, and age-related factors (25, 26, 31). The MET-based intensity thresholds used here may therefore misclassify borderline windows for participants whose true metabolic response departs substantially from the population average. Studies incorporating concurrent indirect calorimetry would provide a more precise reference standard for evaluating energy expenditure classification at the individual level.

Third, all participants were recruited from a single university in China, and the sample was limited to healthy students aged 18 to 25 years. Although LOSO cross-validation strengthens the inference of subject-independent generalization within this cohort, the extent to which the findings transfer to students from different cultural, geographic, or institutional contexts remains to be established. Movement patterns, habitual activity levels, and campus environments may vary across universities and countries in ways that could affect model performance. Broader multi-site validation studies would be needed to assess the generalizability of the framework across diverse student populations.

Taken together, these limitations suggest that the present framework should be regarded as a feasible proof of concept for campus-oriented wearable monitoring rather than a fully validated production system. The controlled protocol, MET-based labeling, and single-site sampling each impose constraints on the scope of the conclusions that can be drawn.

## 5 Conclusion

This study developed and validated a wearable IMU-based framework for daily physical activity recognition and energy expenditure level classification in university students. Using a single waist-worn inertial sensor, the framework achieved 93.8% accuracy and a macro-F1 score of 0.932 for six-class activity recognition, and 89.6% accuracy with a macro-F1 score of 0.883 for three-class energy expenditure level classification, both under leave-one-subject-out cross-validation across 96 participants.

Several findings from this study merit emphasis. The CNN-LSTM model achieved the highest point estimates across both tasks, though its performance advantage over XGBoost did not reach statistical significance after Bonferroni correction, suggesting that feature-based pipelines remain a viable option when computational efficiency or model interpretability is a priority. Classification errors were concentrated between stair-related activities and between adjacent moderate- and vigorous-intensity categories, patterns that are consistent with the biomechanical similarity among these classes and that have been observed in comparable wearable HAR studies. Low-intensity behavior was classified with the highest accuracy, reflecting the distinct signal characteristics of sedentary activities relative to ambulatory and vigorous classes.

The primary contribution of this study lies in providing empirical evidence that addresses three specific questions that prior literature could not answer. It establishes, for the first time, that a single waist-worn IMU can achieve acceptable subject-independent stair discrimination in a university student cohort under strict LOSO validation—a conclusion that could not be drawn from existing multi-sensor studies (10, 17), placement-comparison studies (18), or window-split deep learning benchmarks (21, 22). It quantifies the accuracy trade-off incurred by integrating activity recognition and MET-informed intensity classification within a single-sensor pipeline for this population (93.8% vs. 89.6%), demonstrating that both outputs remain practically useful and showing that this integration had not previously been validated in a student-specific context. And it provides a LOSO-validated performance benchmark for campus-typical activities at a 96-participant scale, confirming that the high accuracies reported for CNN-LSTM and Inception-based models in prior window-split evaluations hold under subject-independent conditions for this specific deployment context. By mapping sensor-detected behaviors onto intensity categories that align with established physical activity guidelines, the framework produces output that is directly relevant to campus health surveillance and individualized exercise feedback, rather than requiring *post hoc* interpretation of activity labels. The use of a single waist-worn sensor, combined with subject-independent validation on a purpose-built student dataset, provides a concrete empirical basis for evaluating the practical feasibility of this approach in educational settings.

## Data Availability

The raw data supporting the conclusions of this article will be made available by the authors, without undue reservation.

## References

[1] Bull FC Al-Ansari SS Biddle S Borodulin K Buman MP Cardon G . World Health Organization 2020 guidelines on physical activity and sedentary behaviour. Br J Sports Med. (2020) 54:1451–62. doi: 10.1136/bjsports-2020-102955, 33239350 PMC7719906

[2] Edelmann D Pfirrmann D Heller S Dietz P Reichel JL Werner AM . Physical activity and sedentary behavior in university students—the role of gender, age, field of study, targeted degree, and study semester. Front Public Health. (2022) 10:821703. doi: 10.3389/fpubh.2022.821703, 35784227 PMC9244168

[3] Deliens T Deforche B De Bourdeaudhuij I Clarys P. Determinants of physical activity and sedentary behavior in university students: a qualitative study using focus group discussions. BMC Public Health. (2015) 15:201. doi: 10.1186/s12889-015-1553-4, 25881120 PMC4349731

[4] Vella CA Nelson MC. Patterns and correlates of sedentary behavior among university students. J Am Coll Heal. (2024) 72:3772–80. doi: 10.1080/07448481.2023.2198020, 37053593 PMC10570395

[5] Savage MJ Magistro D Hennis PJ Donaldson J Healy LC Hunter KA . Determining factors of physical activity and sedentary behaviour in university students during the COVID-19 pandemic: a longitudinal study. PLoS One. (2024) 19:e0298134. doi: 10.1371/journal.pone.0298134 38394147 PMC10889634

[6] Prince SA Adamo KB Hamel ME Hardt J Gorber SC Tremblay M. A comparison of direct versus self-report measures for assessing physical activity in adults: a systematic review. Int J Behav Nutr Phys Act. (2008) 5:56. doi: 10.1186/1479-5868-5-56, 18990237 PMC2588639

[7] Skender S Ose J Chang-Claude J Paskow MJ Brühmann B Siegel EM . Accelerometry and physical activity questionnaires—a systematic review. BMC Public Health. (2016) 16:515. doi: 10.1186/s12889-016-3172-0, 27306667 PMC4910242

[8] Ward DS Evenson KR Vaughn A Rodgers AB Troiano RP. Accelerometer use in physical activity: best practices and research recommendations. Med Sci Sports Exerc. (2005) 37:S582–8. doi: 10.1249/01.mss.0000185292.71933.91, 16294121

[9] Lara OD Labrador MA. A survey on human activity recognition using wearable sensors. IEEE Commun Surveys Tuts. (2013) 15:1192–209. doi: 10.1109/SURV.2012.110112.00192

[10] Attal F Mohammed S Dedabrishvili M Chamroukhi F Oukhellou L Amirat Y. Physical human activity recognition using wearable sensors. Sensors. (2015) 15:31314–38. doi: 10.3390/s151229858, 26690450 PMC4721778

[11] Hammerla NY Halloran S Ploetz T. "Deep, convolutional, and recurrent models for human activity recognition using wearables". In: Proc. 25th Int. Joint Conf. Artif. New York, NY, USA: Intell (2016). p. 1533–40.

[12] Adesida Y Papi E McGregor AH. Exploring the role of wearable technology in sport kinematics and kinetics: a systematic review. Sensors. (2019) 19:1597. doi: 10.3390/s19071597, 30987014 PMC6480145

[13] O'Reilly M Caulfield B Ward T Johnston W Doherty C. Wearable inertial sensor systems for lower limb exercise detection and evaluation: a systematic review. Sports Med. (2018) 48:1221–46. doi: 10.1007/s40279-018-0878-4, 29476427

[14] Swain TA McNarry MA Runacres AWH Mackintosh KA. The role of multi-sensor measurement in the assessment of movement quality: a systematic review. Sports Med. (2023) 53:2477–504. doi: 10.1007/s40279-023-01905-1, 37698766 PMC10687099

[15] Preece SJ Goulermas JY Kenney LPJ Howard D. A comparison of feature extraction methods for the classification of dynamic activities from accelerometer data. IEEE Trans Biomed Eng. (2009) 56:871–9. doi: 10.1109/TBME.2008.2006190, 19272902

[16] Gupta P Dallas T. Feature selection and activity recognition system using a single triaxial accelerometer. IEEE Trans Biomed Eng. (2014) 61:1780–6. doi: 10.1109/TBME.2014.2307069, 24691526

[17] Cleland I Kikhia B Nugent C Boytsov A Hallberg J Synnes K . Optimal placement of accelerometers for the detection of everyday activities. Sensors. (2013) 13:9183–200. doi: 10.3390/s130709183 23867744 PMC3758644

[18] From Wrist to Ankle: Understanding IMU Sensor Placement in Human Activity Recognition. 2025 Emerging Technologies for Intelligent Systems (ETIS). (2025) p. 1–6.

[19] Zhang S Li Y Zhang S Shahabi F Xia S Deng Y . Deep learning in human activity recognition with wearable sensors: a review on advances. Sensors. (2022) 22:1476. doi: 10.3390/s22041476, 35214377 PMC8879042

[20] Wang J Chen Y Hao S Peng X Hu L. Deep learning for sensor-based activity recognition: a survey. Pattern Recogn Lett. (2019) 119:3–11. doi: 10.1016/j.patrec.2018.02.010

[21] Mekruksavanich S Jitpattanakul A. iSPLInception: an inception-ResNet deep learning architecture for human activity recognition. IEEE Access. (2021) 9:68985–9001. doi: 10.1109/ACCESS.2021.3078184

[22] Khatun MA Yousuf MA Ahmed S Uddin MZ Alyami SA Al-Ashhab S . Deep CNN-LSTM with self-attention model for human activity recognition using wearable sensor. IEEE J Transl Eng Health Med. (2022) 10:1–15. doi: 10.1109/JTEHM.2022.3177710, 35795873 PMC9252338

[23] Lopes JM Figueiredo J Fonseca P Cerqueira JJ Vilas-Boas JP Santos CP. Deep learning-based energy expenditure estimation in assisted and non-assisted gait using inertial, EMG, and heart rate wearable sensors. Sensors. (2022) 22:7913. doi: 10.3390/s22207913, 36298264 PMC9607229

[24] Lee CJ Lee JK. IMU-based energy expenditure estimation for various walking conditions using a hybrid CNN–LSTM model. Sensors. (2024) 24:414. doi: 10.3390/s24020414, 38257507 PMC10821340

[25] Leone A Rescio G Diraco G Manni A Siciliano P Caroppo A. Ambient and wearable sensor technologies for energy expenditure quantification of ageing adults. Sensors. (2022) 22:4893. doi: 10.3390/s22134893, 35808387 PMC9269397

[26] Staudenmayer J Zhu D Catellier PS. Statistical considerations in the analysis of accelerometry-based activity monitor data. Med Sci Sports Exerc. (2012) 44:S61–7. doi: 10.1249/MSS.0b013e3182399e0f, 22157776

[27] Khan AM Lee Y-K Lee SY Kim T-S. A triaxial accelerometer-based physical-activity recognition via augmented-signal features and a hierarchical recognizer. IEEE Trans Inf Technol Biomed. (2010) 14:1166–72. doi: 10.1109/TITB.2010.2051955, 20529753

[28] Ainsworth BE Haskell WL Herrmann SD Meckes N Bassett DR Jr Tudor-Locke C . 2011 compendium of physical activities: a second update of codes and MET values. Med Sci Sports Exerc. (2011) 43:1575–81. doi: 10.1249/MSS.0b013e31821ece12, 21681120

[29] Butterworth S. On the theory of filter amplifiers. Exp Wirel Wirel Eng. (1930) 7:536–41.

[30] Chen T Guestrin C. "XGBoost: a scalable tree boosting system". In: Proc. 22nd ACM SIGKDD Int. Conf. Knowl. Discov. New York, NY, USA: Data Min (2016). p. 785–94. doi: 10.1145/2939672.2939785

[31] Montoye AHK Pivarnik JM Mudd LM Biswas S Pfeiffer KA. Evaluation of the activPAL accelerometer for physical activity and energy expenditure estimation in a semi-structured setting. J Sci Med Sport. (2017) 20:1003–7. doi: 10.1016/j.jsams.2017.04.011, 28483558

